# Stigmatizing Policies Interact with Mental Health and Sexual Behaviours to Structurally Induce HIV Diagnoses Among European Men Who Have Sex with Men

**DOI:** 10.1007/s10461-022-03683-9

**Published:** 2022-04-17

**Authors:** Kristefer Stojanovski, Elizabeth J. King, K. Rivet Amico, Marisa C. Eisenberg, Arline T. Geronimus, Sladjana Baros, Axel J. Schmidt

**Affiliations:** 1 Department of Social, Behavioral and Population Sciences, School of Public Health and Tropical Medicine, Tulane University, New Orleans, LA USA; 2 Department of Health Behavior and Health Education, School of Public Health, University of Michigan, Ann Arbor, MI USA; 3 Department of Epidemiology, School of Public Health, University of Michigan, Ann Arbor, MI USA; 4 Department of Mathematics, College of Literature, Science, and the Arts, University of Michigan, Ann Arbor, MI USA; 5 Population Studies Centre, Institute for Social Research, University of Michigan, Ann Arbor, MI USA; 6 Department for HIV, STI, Viral Hepatitis, and Tuberculosis, Institute of Public Health of Serbia “Dr Milan Jovanovic Batut”, Belgrade, Serbia; 7 Sigma Research, Department of Public Health, Environments and Society, London School of Hygiene and Tropical Medicine, London, UK

**Keywords:** MSM, Europe, Policy, Stigma, HIV, Structural determinants

## Abstract

Structural stigma shapes men who have sex with men’s (MSM’s) mental health and sexual behaviours. The aim of this study was to examine how stigmatizing policies interact with downstream anxiety/depression and sexual behaviours to structurally pattern HIV disparities among European MSM. We conducted a secondary data analysis of the European Men-who-have-sex-with-men Internet Survey (EMIS) from 2017. We included a total of 98,600 participants living in 39 European countries. We used the Rainbow Index, a score given to countries based on their sexual and gender minority policies as the predictor of HIV diagnosis. We conducted adjusted random intercept and slope multi-level logistic regressions. In adjusted models, higher Rainbow Index scores was associated with lower predictive probabilities of diagnosed HIV, regardless of the number of condomless intercourse partners. The predictive probability of HIV diagnosis was also lower, regardless of severity of anxiety/depression, where the Rainbow Index score was better. Country-level policies interact with downstream sexual behaviours and anxiety/depression to structurally influence HIV diagnosis among MSM in Europe.

## Introduction

Population-level inequities in HIV exist globally, despite advances in prevention and treatment. In the World Health Organization (WHO) European Region, which extends into Central Asia, HIV incidence increased by 9% from 2010 to 2019 [1]. The increase is largely driven by trends in WHO Eastern and Central European countries. In WHO Eastern Europe, the incidence rate increased by 23% from 2010 to 2019 (33.9 per 100,000 in 2010 to 41.7 in 2019) [1]. In WHO Central Europe, the incidence rate increased by 113% from 1.6 cases per 100,000 to 3.4 cases per 100,000 in the same time period [1]. In WHO Western Europe, incidence decreased by 24% from 7.5 cases per 100,000 people to 5.7 cases per 100,000 people from 2010 to 2019 [1].

Men who have sex with men (MSM) in Europe experience more HIV inequities. Male-to-male sexual contact is the second highest transmission route in the European continent [1]. Variation exists depending on the region or country in which MSM reside. In the European Economic Area, sex between men is the main route of HIV transmission, accounting for 39% of all diagnosed HIV cases in 2019 [1]. Sex between men accounted for more than 60% of diagnosed HIV cases in 10 countries—Croatia, Czechia, Germany, Hungary, Iceland, the Netherlands, Poland, Slovakia, Slovenia and Spain—when the mode of transmission was known in 2019 [1]. There were large increases in Bulgaria, Cyprus, Estonia, Lithuania, Poland, Romania and Slovakia in recent years [1]. These statistics portray the higher risk of HIV infection that MSM contend with in Europe.

According to Erving Goffman, “Society establishes the means of categorizing persons and the complement of attributes felt to be ordinary and natural for members of each of these categories” [2]. Stigma aims to categorize, label, and separate people as different and shapes the lived experiences of those being stigmatized. Stigma is posited as a fundamental cause of poor health because it influences many diseases through multiple risk factors, it involves access and distribution of power and resources that can help mitigate risks or disease, and is related to health inequities over time and space [3, 4]. Stigma can operate across the socioecological model, such as structurally through policies, institutionally by values and norms, interpersonally in day to day interactions, and individually through internalized homophobia [5]. In one European-wide study, anti-gay and anti-immigrant stigma in the countries to which MSM migrated was associated with reduced knowledge about prevention and condom usage [6]. A study in Barcelona (Spain), Bratislava (Slovakia), Bucharest (Romania), Ljubljana (Slovenia), Prague (Czechia), and Verona (Italy) found that gay-related stigma was associated with elevated odds of sex under the influence of alcohol, cannabis, and other substances [7]. In the same study, a one standard deviation increase in stigma was associated with an 11% higher odds of condomless sex [7]. A study in 14 European countries found that people living with HIV (PLHIV) who experienced discrimination in healthcare settings had more prevention needs, such as safer sex practices, communicating with partners about sexuality, and prevention of STIs [8]. The European Centres for Disease Prevention and Control (ECDC) found that two-thirds of government health representatives in the WHO European region reported stigma among healthcare professionals perpetrated against key populations, such as MSM, serve as barriers to HIV testing, treatment, and prevention services and contribute to late HIV diagnoses [9]. These diverse geographic studies indicate that stigma plays a role in the social patterning of HIV among European MSM.

Stigma also shapes intermediary factors to HIV risk, such as mental health. Global research portrays how MSM’s mental health inequities are socially patterned due to stigmatization [10–15]. For example, MSM in St. Petersburg, Russia, surveyed after the passage of the local anti-gay “propaganda” ordinance (March 2012) had a 1.7-fold greater likelihood of depression, as compared to MSM surveyed before the ordinance [16]. Among MSM in this study who experienced stigma, depression was three times greater [AOR = 2.92, 95% CI (2.02–4.24)] [16]. A 48 country study uncovered that living in a highly stigmatizing policy context (as compared to not) was associated with elevated depression (adjusted β = 0.16, 95% CI [0.07, 0.25] and living in a country with more protective policies for longer further reduced depression (adjusted β = − 0.33, 95% CI [− 0.49, − 0.17] [17]. Research also links poor mental health to HIV risk by reducing the likelihood of condom use during sex, increasing utilization of substances, and elevating numbers of sexual partners [6, 18, 19], all well known risk factors for HIV transmission. A study in France, Germany, Italy, and the United Kingdom indicated that depression was associated with reduced adherence to HIV treatment among PLHIV [20]. A meta-analysis of 39 studies, reported significant associations between depression and lower odds of retention in HIV care among PLHIV [21]. As described, stigma influences mental health, which itself can influence HIV diagnosis, thus requiring greater study of the interplay between structural stigma, mental health, and HIV diagnosis.

Structural stigma, in the form of policies, may play an upstream role in shaping HIV risk. Policies structure the lives of MSM by supporting or not the right for MSM to marry, protecting against discrimination, enshrining access to services, and influencing mental health. While stigma is a major factor influencing the mental well-being and risk for HIV among MSM in Europe, further research is needed to identify the stigmatizing structural pathways that shape HIV diagnosis. We aim to estimate the extent to which LGBTQ + country-level policy variations interact with downstream health behaviours and anxiety/depression to influence HIV prevalence among European MSM. We hypothesize that stigmatizing policies will positively interact with mental health and sexual behaviours to elevate the probability of HIV diagnoses.

## Materials and Methods

We used data from the anonymous 2017 European Men-Who-Have-Sex-with-Men Internet Survey (EMIS-2017) administered from October 2017 to January 2018 in 50 predominantly European countries (www.emis2017.eu). The questionnaire had 409 items in the following areas: demographics; morbidities (including violence/abuse and mental health); sexual and drug-using behaviours, including HIV testing and condom use; unmet needs; and knowledge and utilization of interventions. The surveys were self-completed, and all data are self-reported. The Observational Research Ethics Committee at the London School of Hygiene and Tropical Medicine approved the study (reference 14421/RR/8805).

### Survey Administration and Sampling

EMIS-2017 promoted the survey on geo-spatial sexual applications (e.g., PlanetRomeo, Grindr), national and trans-national commercial, and non-governmental websites; and social networking sites (e.g., Facebook). The survey and sampling methodology has been previously published [22].

A total of 126,261 MSM participated in 42 European countries. We excluded Albania, Kosovo^1^ and Montenegro because sample sizes were less than 100. The analytic sample includes 126,090 MSM living in 39 countries and four microstates (embedded within countries). Table 1 outlines the countries included in our analyses. The survey was translated into 23 out of the 24 official European Union languages (excluding Gaelic Irish, estimated at 170,000 speakers) and six other European languages (Albanian, Norwegian, Macedonian, Russian, Turkish, and Ukrainian). The survey was translated into Arabic, the predominant language of migrants into Europe since 2014. The survey was pretested in all the languages by non-governmental partners. The University of Michigan Health and Behavioural Sciences Institutional Review Board categorized this secondary analysis as exempt (HUM00165101).

**Table 1:** European countries and full sample sizes included in EMIS-2017 (n = 126,090)

Austria (n = 2705)	Bosnia & Herzegovina (n = 232)	Belarus (n = 3038)	Belgium (n = 1177)
Bulgaria (n = 440)	Croatia (n = 1015)	Cyprus (n = 307)	Czechia (n = 1897)
Denmark (n = 1698)	Estonia (n = 212)	Finland (n = 1409)	France* (n = 10,996)
Germany (n = 23,107)	Greece (n = 2909)	Hungary (n = 1015)	Iceland (n = 111)
Ireland (n = 2083)	Italy* (n = 11,025)	Latvia (n = 370)	Lithuania (n = 169)
Luxembourg (n = 252)	Malta (n = 299)	Moldova (n = 498)	Netherlands (n = 3851)
North Macedonia (n = 175)	Norway (n = 2957)	Poland (n = 4025)	Portugal (n = 2555)
Romania (n = 2002)	Russia (n = 6247)	Serbia (n = 1041)	Slovakia (n = 1003)
Slovenia (n = 685)	Spain* (n = 10,652)	Sweden (n = 4443)	Switzerland* (n = 3383)
Turkey (n = 1855)	Ukraine (n = 1201)	United Kingdom (n = 11,889)	

*Includes microstates: Monaco (France), San Marino (Italy), Liechtenstein (Switzerland), and Andorra (Spain)

### Variables

#### Outcome Variables

The primary outcome was (self-reported) HIV diagnosis (n = 13,059). We excluded persons who reported never receiving an HIV test (n = 26,641). Participants who reported their last HIV test was negative were considered to have a negative HIV status (n = 85,541). Our final sample size was 98,600. We created a second outcome variable for a sensitivity analysis that aimed to better estimate the HIV negative status by excluding participants who tested HIV negative longer than 12 months ago (n = 23,223); the total sample size for this outcome was 75,295.

#### Explanatory Variables

We used the Rainbow Index to assess country-level stigmatizing policies developed by the International Lesbian, Gay, Bisexual, Transgender, and Intersex Association of Europe. The index ranks countries based on their policies that afford rights and protections to lesbian, gay, bisexual, transgender, and queer (LGBTQ +) persons [23]. The Rainbow Index includes policies such as the right to marriage, anti-discrimination legislation, to name a couple. We used the Rainbow Index from 2017 to match the year of the data collection. For 2017, the Rainbow Index ranged from 6 to 88 (theoretical range 0–100).

We included two main explanatory variables. The first was a categorically combined anxiety/depression variable that collapsed scores from the Patient Health Questionnaire (PHQ-4) [24]. The categories were “normal,” “mild,” “moderate,” and “severe.” The PHQ-4 has been validated in Germany, the PHQ-9 in Latvia, for both Latvian and Russian languages, and in Spain, and the PHQ-2 in Latvia as well [25–27]. However, the use of the PHQ-4 may pose challenges in other cultural contexts and may not fully capture mental health effects, as compared to longer item scales. The second explanatory variable was the number of non-steady male condomless intercourse partners in the last year. This variable was re-coded to an ordinal scale (0, 1–10, 11–20, …, 51 +).

#### Covariates

We included six confounder variables. The first was financial coping (“Which of these phrases would you say comes closest to your feelings about your income these days?”), which was a 5-point Likert scale that ranged from one (“living really comfortably on present income”) to five (“really struggling on present income”). Secondly, sexual identity, which was recoded as gay/homosexual, bisexual, or other. Thirdly, education, which was reported as years of formal education after 16 years of age. Fourthly, an outness variable (“Thinking about all the people who know you (including family, friends and work or study colleagues), what proportion know that you are attracted to men?”) categorized into three levels: “out to none/few”, “out to some,” and “out to (almost) all”. The fifth covariate was count of three questions on the recency of homophobic intimidation, verbal insults, or physical violence. If respondents ever experienced the events they were categorized as a one, thus the total range was from zero (never experienced any) to three (ever experienced intimidation, verbal insults, and physical abuse) [28, 29]. Last was age, categorized as: less than 25 years of age, 25–39 years, and 40 years or more. We controlled for where recruitment occurred (i.e., centrally via dating apps or locally through NGOs) and multi-discrepancy in answers.

### Statistical Analyses

We used univariate frequencies, and percentages to examine sociodemographics, depression/anxiety, condomless anal sex, HIV diagnosis, and geographic areas of residence in Europe. Next, we conducted bivariate analyses to understand the associations between sociodemographic, explanatory variables, and confounders with the outcome of HIV diagnosis using Pearson chi-square measures of association (p < 0.05 was used to determine statistical significance).

The data had a nested structure of participants within countries and cities. We could not obtain within country regional data due to privacy considerations, and limited data collected in the survey. A variable was available that measured the city size. The level-two variable was the city size, which was nested under the level-three variable of country. By nesting the city size within the country, this acts as a proxy for major cities and towns within countries. City size was an important variable to include, given that one’s city size may alter the social networks of MSM, including potential number of sexual partners, and exposure to HIV. Research documents geographic variation in sexual orientation by city size (i.e., more gay men in larger cities) [30]. City size was as follows: (1) a very big city or town (a million or more people); (2) a big city or town (500,000–999,999 people); (3) a medium-sized city or town (100,000–499,999 people); (4) a small city or town (10,000–99,999 people); and (5) a village or the countryside (less than 10,000 people).

Given the nested nature of the data of participants (level-one) within cities of a certain size (level-two) within countries (level-three), we used a random intercept and slope multilevel logistic regression model to test the association between the Rainbow Index of the country and an individual’s binary HIV diagnosis outcome. The level-one variables included the explanatory variables, other covariates, and the outcome variable of HIV diagnoses. We created two multi-level models per outcome. The first was a basic model with each covariate or explanatory variable serving as its own variable in the model. In the second, we created an interaction term between the Rainbow Index (a level-3 variable) and two downstream explanatory variables, anxiety/depression and condomless anal sex with non-steady partners (level one variables). We adjusted for all covariates described above because they were significant in bivariate analyses (p < 0.05) and have been identified as salient factors in the research literature. We report adjusted odds ratios (OR) and corresponding 95% confidence intervals (CI) from the multi-level logistic regression models.

The main analyses of interest from the multi-level models were the predictive probabilities. The predictive probabilities estimate the chance of reporting an HIV diagnosis based on all the variables within the model*.* For these analyses, we graphed the predictive probabilities of HIV diagnosis by the Rainbow Index and their interaction with anxiety/depression and condomless anal sex with non-steady partners, while also adjusting for other variables. We analysed the data using Stata 16 [31].

While the total sample size for the main outcome was 98,600, the analytic sample varied according to the missingness of the explanatory variables and covariates. A complete case methodology was used for the analyses, in line with other EMIS research [6]. The condomless non-steady intercourse partners variable was missing for 4.5% of the sample (n = 4389) and anxiety/depression were missing for about 1.4% (n = 1412). The education variable had 7.2% (n = 9081) missing and experiences of abuse variables had 0.6% (n = 749) missing. The outness and the city size variables had 1% missing (n = 1027 and 1095, respectively). The sexual orientation and financial coping variables had less that 1% missing (n = 76 and n = 340, respectively). Using a complete case analysis approach, the analytic sample for the multivariable multi-level models of HIV diagnosis was reduced to 85,209 and for the sensitivity analyses it was reduced to 65,189.

## Results

### Geographic Findings

Of the 98,600 participants, the majority (85.5%, n = 84,294) were living in European Union countries. Participants in the European Free Trade Association states of Iceland, Liechtenstein, Norway, Switzerland accounted for 5.3% (n = 5250) of the sample. Participants from Russia made up 5.4% (n = 5307) of the sample. The Eastern European Neighbourhood Policy countries—Belarus, Moldova, and Ukraine accounted for 1.7% (n = 1657) of the sample. Lastly, 2.1% (n = 2092) of participants lived in EU Enlargement area states—Bosnia & Herzegovina, North Macedonia, Serbia, and Turkey. The average Rainbow Index score was 50.8 (Stdev = 21.5, Range: 6–88).

### Sociodemographic Results

The percentage of the sample under 25 years of age was 13% (n = 12,638), 46% (n = 45,254) were 25–39 years of age, and 41% (n = 40,708) were 40 years of age or older. Most respondents, 81% (n = 80,146) identified as gay/homosexual, 13% (n = 12,686) as bisexual, 6% (n = 5692) as other, which included 1% stating straight/heterosexual (Table 2). There was variation in time spent in formal education past age 16 as outlined in Table 2. In terms of financial coping, 14% (n = 13,532) stated they were really comfortable, 37% (n = 36,647) comfortable, 33% (n = 32,394) neither comfortable nor uncomfortable, 12% (n = 11,350) were struggling, and 4% (n = 4337) really struggling on current income. All sociodemographic variables were statistically significant and associated with HIV diagnosis in bivariate analyses (Table 2).

**Table 2:** Frequencies and percentages of sociodemographic characteristics and unadjusted chi-square measures of the associations between sociodemographics and HIV diagnosis (n = 98,600)

		Ever diagnosed with HIV (n = 98,600)		Test statistic*	p-value
	N (%)	Tested & Positive, N (%)	Tested & Negative, N (%)		
Education past 16 years of age					
None	3009 (3.3)	560 (4.6)	2449 (3.1)	χ^2^ = 152.7	< 0.001
1 year	1145 (1.2)	162 (1.3)	983 (1.2)		
2 years	4923 (5.3)	733 (6.0)	4190 (5.2)		
3 years	7209 (7.8)	1021 (8.4)	6188 (7.7)		
4 years	8086 (8.8)	1071 (8.8)	7015 (8.8)		
5 years	10,809 (11.7)	1473 (12.1)	9336 (11.7)		
6 years	10,638 (11.5)	1386 (11.4)	9252 (11.6)		
7 years	9284 (10.1)	1145 (9.4)	8139 (10.2)		
8 years	10,379 (11.3)	1255 (10.3)	9124 (11.4)		
9 years	5245 (5.9)	582 (4.8)	4843 (6.1)		
10 years	7455 (8.1)	904 (7.4)	6551 (8.2)		
More than 10 years	13,802 (15.0)	1863 (15.3)	11,939 (14.9)		
Financial coping					
Living really comfortably	13,532 (13.8)	1681 (12.9)	11,851 (13.9)	χ^2^ = 112.0	< 0.001
Living comfortably	36,647 (37.3)	4641 (35.7)	32,006 (37.5)		
Neither comfortable nor struggling	32,394 (33.0)	4230 (32.5)	28,164 (33.0)		
Struggling	11,350 (11.6)	1709 (13.1)	9641 (11.3)		
Really struggling	4337 (4.4)	747 (5.7)	3590 (4.2)		
Sexual identity					
Gay	80,146 (81.4)	11,686 (89.6)	68,460 (80.1)	χ^2^ = 703.2	< 0.001
Bisexual	12,686 (12.9)	830 (6.4)	11,856 (13.9)		
Other	5692 (5.8)	522 (4.0)	5170 (6.1)		

*Pearson chi-square statistic

### Explanatory Variable Results

Most of the respondents were out to “all or almost all”, 46% (n = 44,744), 29% (n = 28,482) were out to some, and 25% (n = 24,347) were out to “none” or “few” (Table 3). Most persons (49%, n = 47,126) were categorised as ‘normal’ on the PHQ-4, 34% (n = 32,906) ‘mild’, 10% (n = 10,099) ‘moderate’, and 7% (n = 7057) ‘severe.’ The majority (57%, n = 53,936) of participants reported zero condomless intercourse male partners in the previous 12 months, while 35% (n = 33,294) stated they had intercourse with 1–10 non-steady male partners without a condom, 4% (n = 3337) reported 11–20, 2% (n = 1401) stated 21–30, 0.6% (n = 583) reported 31–40, 0.4% (n = 357) said 41–50, and 1·4% (n = 1303) had more than 50 non-steady partners without a condom. All explanatory variables were statistically significant in their associations with HIV diagnosis in bivariate analyses (Table 3).

**Table 3:** Frequencies and percentages of explanatory variables and unadjusted chi-square measures of association of explanatory variables and HIV diagnosis (n = 98,600)

	N (%)	Ever diagnosed with HIV (n = 98,600)		Test statistic*	p-value
		Tested & positive N (%)	Tested & negative N (%)		
Country’s rainbow index					
Low (poor policies)	28,332 (29.7)	3647 (28.4)	24,685 (29.9)	χ^2^ = 31.6	< 0.001
Medium	25,547 (26.8)	3303 (25.8)	22,244 (26.9)		
High (good policies)	41,510 (43.5)	5872 (45.8)	35,638 (43.2)		
Number of condomless non-steady male intercourse partners, previous 12 months					
None	53,936 (57.3)	4207 (34.0)	49,729 (60.8)	χ^2^ = 8000.0	< 0.001
1–10	33,294 (35.3)	5034 (40.6)	28,260 (34.5)		
11–20	3337 (3.5)	1299 (10.5)	2038 (2.5)		
21–30	1401 (1.5)	648 (5.2)	753 (0.9)		
31–40	583 (0.6)	298 (2.4)	285 (0.4)		
41–50	357 (0.4)	176 (1.4)	181 (0.2)		
51 +	1303 (1.4)	727 (5.9)	576 (0.7)		
Patient health questionnaire-4					
Normal	47,126 (48.5)	6015 (46.9)	41,111 (48.7)	χ^2^ = 21.1	< 0.001
Mild depression/anxiety	32,906 (33.9)	4404 (34.3)	28,502 (33.8)		
Moderate depression/anxiety	10,099 (10.4)	1392 (10.9)	8707 (10.3)		
Severe depression/anxiety	7057 (7.3)	1018 (7.9)	6039 (7.2)		
Experiences of abuse^#^					
None	30,686 (31.1)	3788 (29.0)	26,898 (31.4)	χ^2^ = 179.3	< 0.001
1	16,945 (17.2)	2096 (16.1)	14,849 (17.4)		
2	34,745 (35.2)	4507 (34.5)	30,328 (35.4)		
3	16,224 (16.5)	2668 (20.4)	13,556 (15.9)		
Outness level^					
Out to none or few	24,347 (25.0)	2227 (17.2)	22,120 (26.1)	χ^2^ = 707.9	< 0.001
Out to some	28,482 (29.2)	3506 (27.0)	24,976 (29.5)		
Out to (almost) all	44,744 (45.9)	7234 (55.8)	37,510 (44.3)		

*Pearson chi-square statistic

^#^Experiences of abuse was a count variable of three questions on the recency of homophobic intimidation, verbal insults, or physical violence. If respondents ever experienced the abuse, they were categorized as one for a total possible range from zero (never experienced any) to three (ever experienced intimidation, verbal insults, and physical abuse)

^^^Thinking about all the people who know you (including family, friends and work or study colleagues), what proportion know that you are attracted to men?

### Country-level Stigma, Condomless Anal Sex, and HIV Diagnosis

Among the 98,600 who reported ever having received an HIV test result, 13% (n = 13,059) reported an HIV positive test result, while 87% (n = 85,541) reported a negative HIV test result at their last test. The adjusted multilevel model with no interaction terms (adjusted for age, sexual identity, education, financial coping, abuse, mental health, outness, and the number of condomless intercourse partners) indicated that for every one-unit improvement in the Rainbow Index, the odds of HIV diagnosis was 0.98 (95% CI (0.98, 0.99), Log-likelihood = − 28,412.6, Wald χ^2^ = 7242.5), indicating that more protective policies reduced the odds of reporting an HIV diagnosis, although the effect was small (Table 4).

**Table 4:** Adjusted odds ratios and 95% confidences intervals of HIV diagnosis from multi-level model by condomless anal sex partners and Patient Health Questionnaire-4 explanatory variables with no interaction terms (n = 85,209)

	Ever diagnosed with HIV*, #	
	OR	95% CI
Country’s rainbow index	0.98	(0.98, 0.99)
Number of condomless non-steady male intercourse partners, previous 12 months (none = reference)		
1–10 partners	2.19	(2.09, 2.30)
11–20 partners	7.03	(6.47, 7.65)
21–30 partners	9.89	(8.77, 11.16)
31–40 partners	11.31	(9.41, 13.59)
41–50 partners	10.91	(8.68, 13.71)
51 + partners	14.48	(12.76, 16.43)
Patient health questionnaire-4 (normal = reference)		
Mild depression/anxiety	1.13	(1.07, 1.19)
Moderate depression/anxiety	1.23	(1.14, 1.32)
Severe depression/anxiety	1.32	(1.21, 1.44)
City size (level-2)	0.03	(0.02, 0.05)
Country (level-3)	0.13	(0.07, 0.22)

*Controlled for outness, experiences of abuse, age, financial coping, formal education past 16 years of age, sexuality of respondent, type of recruitment (local vs. central), and multiple discrepancies in answers

^#^Model test statistics: log-likelihood = − 28,412.6, Wald χ^2^ = 7242.5, p < 0.001

**Fig. 1:**
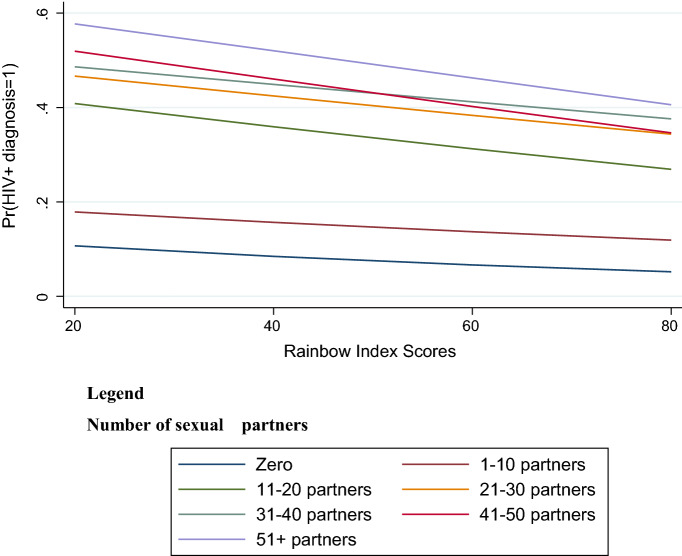
Predictive probability of HIV diagnosis in national samples by Rainbow Index Scores and number of condomless non-steady male sex partners (n = 85,209). [Pr(HIV + diagnosis = 1)] is the probability of HIV diagnosis. Model test statistics: Log-likelihood = − 28,387.1, Wald χ^2^ = 7260.6, p < 0.000

In the multivariable interaction models, the predictive probability (the main analysis of interest) of HIV diagnosis changed with the Rainbow Index score of the country in which MSM resided across the two explanatory variables of interest. With higher Rainbow Index scores (better policies), the predictive probability of an HIV diagnosis was lower, regardless of the number of non-steady partners in which a condom was not used (Fig. 1). However, MSM with a lower number of partners had lower starting predictive probabilities of HIV diagnosis, which got lower with higher Rainbow Index scores. The slopes also varied by the number of condomless non-steady male intercourse partners, such that the predictive probability of HIV diagnosis was lower for MSM with a higher number of condomless partners. For example, participants with 51 + condomless partners in the last year (purple line in Fig. 1) who lived in a country with a Rainbow Index score of 20 (low protections) would have a 60% chance of having HIV. However, if the same participant lived in a country with a Rainbow Index score of 80 (high protections) the probability of HIV was 40% (Log-likelihood = − 28,387.1, Wald χ^2^ = 7260.6).

**Fig. 2:**
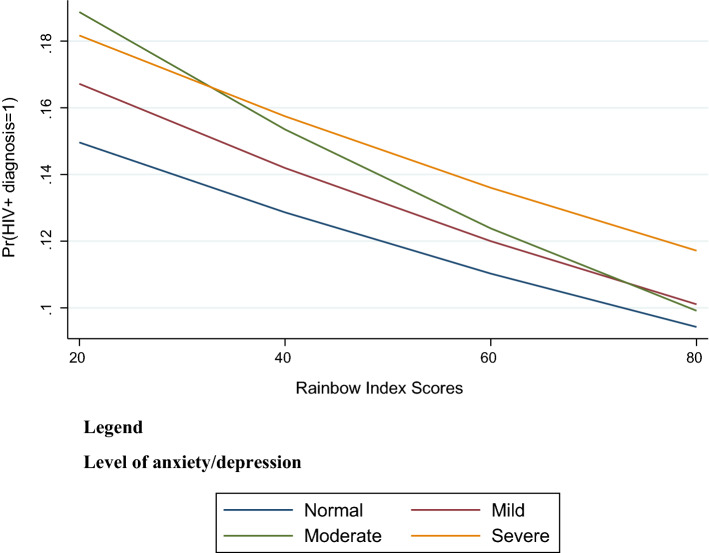
Predictive probability of HIV diagnosis in national samples by Rainbow Index Scores and anxiety/depression (n = 85,209). [Pr(HIV + diagnosis = 1)] is the probability of HIV diagnosis. Model test statistics: Log-likelihood = − 25,774.0, Wald χ^2^ = 6149.3, p < 0.000

The analyses showed similar trends for anxiety/depression. The predictive probability of an HIV diagnosis was lower with higher Rainbow Index scores across all levels of anxiety/depression. The predictive probability of an HIV diagnosis was lowest among participants who did not report anxiety/depression. MSM with ‘severe’ anxiety/depression experienced a smaller change in the probability of HIV diagnosis as the Rainbow Index score got higher, although its initial predictive probability value was lower (Fig. 2). For example, participants with ‘severe’ anxiety/depression in a country with a Rainbow Index score of 20 (low protections) would have a 19% chance of diagnosed HIV, while in a country with Rainbow Index score of 80 (high protections) they would have a 12% chance (Log-likelihood = − 25,774.0, Wald χ^2^ = 6149.3).

### Sensitivity Analyses

The sensitivity analysis that aimed to better approximate HIV negative status indicates that the relationship between the number of condomless sexual partners and anxiety/depression had a similar association with HIV diagnosis. For condomless sexual partners, the predictive probabilities were the same. However, for anxiety/depression the predictive probabilities were higher by 5–7%.

## Discussion

The analysis showed that European MSM living in countries with more protective national policies for LGBTQ + persons had lower predictive probabilities of HIV diagnoses, even when individual-level risk factors for HIV were present. These findings provide evidence that policies can act as a significant structural determinants of HIV risk in Europe.

Globally, the structural determinants of health are critical points of intervention to improve HIV prevention. Research has shown how stigmatizing contexts can hamper HIV prevention efforts and risk behaviours. Pachankis et al. found that structural stigma toward sexual minority immigrants in Europe was associated with lower HIV-prevention knowledge, service coverage, and risk-reducing behaviours among migrants [6]. A multi-country study by Arreola et al. indicated that participants living in countries where same-sex behaviours are criminalized, as compared to not, had reduced access to prevention services and HIV treatment [32]. The results of our study indicate that by crafting supportive LGBTQ + policies, the higher order intervention (policy improvement) can drastically reshape HIV prevention efforts in Europe by reducing its influence on other downstream “risk” factors (i.e., sexual behaviors and mental health).

As our analysis showed, laws and policies are important factors to explore in the study of population HIV disparities. Exploring how policies may influence HIV risk and infection via intermediate determinants is a substantial gap in the research literature. A recent global study examined HIV policy alignment with global norms (e.g., guidelines about immediate ARV treatment for PLHIV, non-discrimination protections) and the influence on HIV risk [33]. Kavanagh et al. found that from 2010 to 2019, new HIV infections fell by 38% in southern Africa, while increasing by 72% in Eastern Europe and Central Asia (EECA) [33]. Many countries in southern Africa have aligned their policies with global prevention norms (e.g., non-discrimination protections, immediate ART, national human rights institutions) [33]. While only a few countries’ policies in EECA have done the same, which hampers HIV testing, access and utilization of services and thus structurally inducing HIV risk and diagnoses [33]. In certain Western European nations, for example, France, Italy, and the United Kingdom there have been greater policy adoptions aligned with international standards [33]. This article adds to the global policy literature by estimating how LGBTQ + rights and policies, or lack thereof, can influence HIV diagnosis by shaping intermediary processes.

As with all studies, limitations to the analyses exist. Firstly, the study is cross-sectional, which hinders the capacity to make causal inferences and the causal chain could be backwards or circular (e.g., HIV diagnosis worsens mental health). We included critical confounding variables that were associated with HIV diagnoses in bivariate analyses to mitigate this limitation. However, it seems challenging to invoke reverse causation that HIV diagnosis or number of condomless intercourse partners influence the Rainbow Index, which measures complicated policy-making processes. Secondly, as EMIS-2017 was an online survey, all data, including HIV diagnosis, are self-reported, possibly leading to an underestimation of the main outcome for those infected with HIV but not yet diagnosed. We addressed this limitation by excluding untested men, and—in sensitivity analysis—men not tested for HIV in the previous 12 months. Because of the anonymous nature of the survey, we believe that social desirability bias plays less of a role for this analysis, as compared to HIV disclosures in healthcare settings or in-person; and studies suggest that self-report may be a reliable measure of HIV [34, 35]. Thirdly, given that recruitment occurred by geospatial dating apps, NGOs, and social media, and that data collection occurred online, selection bias is a concern, and the results may not be generalizable outside the study population. Selection bias may have inadvertently found MSM more connected to the internet given the use of an internet survey and thus may have more information about and use of services. Additional research is needed to assess these relationships in different cultural contexts where stigmatizing norms and process may vary [16, 36–39]. Fourthly, the large sample size would be able to detect significant findings, which may not necessarily be epidemiologically relevant (as seen in some of our analyses where the odds ratios were small). However, the main analyses are the predictive probabilities, rather than each variable’s odds ratios. The sample also had larger representation from the Western and Northern European geographies, as compared to Eastern Europe. Greater recruitment in Eastern Europe might strengthen the associations found in this study given the growing rates of HIV. Lastly, given that no personal identifiers, including IP addresses, were collected it is possible that persons could take the survey twice. However, no stipends were used, and the questionnaire was long thus reducing the incentive to duplicate responses. Despite these limitations, this study adds to the global literature portraying how structural policy determinants play upstream roles in shaping HIV diagnosis.

## Conclusion

Policies interact with downstream social determinants to elevate the probability of HIV diagnosis among European MSM. Supportive policy environments can reduce the probability of living with HIV. Given the interactional relationship between policies, condomless intercourse, and anxiety/depression, systems science conceptualizations of HIV risk that further explore the role of policies could enhance HIV prevention and research efforts.

## Data Availability

Data are not available given General Data Protection Regulations of the European Union.

## Footnotes

1 *References to Kosovo shall be understood to be in the context of United Nations Security Council Resolution 1244 (1999).*

## Code Availability

The coding for statistical analysis is available to review upon request.
