# Correction to: Stigmatizing Policies Interact with Mental Health and Sexual Behaviours to Structurally Induce HIV Diagnoses Among European Men Who Have Sex with Men

**DOI:** 10.1007/s10461-022-03884-2

**Published:** 2022-10-18

**Authors:** Kristefer Stojanovski, Elizabeth J. King, K. Rivet Amico, Marisa C. Eisenberg, Arline T. Geronimus, Sladjana Baros, Axel J. Schmidt

**Affiliations:** 1 Department of Social, Behavioral and Population Sciences, School of Public Health and Tropical Medicine, Tulane University, New Orleans, LA USA; 2 Department of Health Behavior and Health Education, School of Public Health, University of Michigan, Ann Arbor, MI USA; 3 Department of Epidemiology, School of Public Health, University of Michigan, Ann Arbor, MI USA; 4 Department of Mathematics, College of Literature, Science, and the Arts, University of Michigan, Ann Arbor, MI USA; 5 Population Studies Centre, Institute for Social Research, University of Michigan, Ann Arbor, MI USA; 6 Department for HIV, STI, Viral Hepatitis, and Tuberculosis, Institute of Public Health of Serbia “Dr Milan Jovanovic Batut”, Belgrade, Serbia; 7 Sigma Research, Department of Public Health, Environments and Society, London School of Hygiene and Tropical Medicine, London, UK

## Correction to: AIDS and Behavior (2022) 26:3400–3410 10.1007/s10461-022-03683-9

The article “Stigmatizing Policies Interact with Mental Health and Sexual Behaviours to Structurally Induce HIV Diagnoses Among European Men Who Have Sex with Men” written by Kristefer Stojanovski, Elizabeth J. King, K. Rivet Amico, Marisa C. Eisenberg, Arline T. Geronimus, Sladjana Baros and Axel J. Schmidt was originally published electronically on the publisher’s internet portal on 17th April 2022 without open access. With the author(s)’ decision to opt for Open Choice the copyright of the article changed on 26th September 2022 to © The Author’s 2022 and the article is forthwith distributed under a Creative Commons Attribution.

The original article has been corrected.

